# EFFECTS OF MOTOR IMAGERY-BASED NEUROFEEDBACK TRAINING AFTER BILATERAL REPETITIVE TRANSCRANIAL MAGNETIC STIMULATION ON POST-STROKE UPPER LIMB MOTOR FUNCTION: AN EXPLORATORY CROSSOVER CLINICAL TRIAL

**DOI:** 10.2340/jrm.v56.18253

**Published:** 2024-03-07

**Authors:** Francisco José SÁNCHEZ-CUESTA, Yeray GONZÁLEZ-ZAMORANO, Marcos MORENO-VERDÚ, Athanasios VOURVOPOULOS, Ignacio J. SERRANO, María Dolores DEL CASTILLO, Patricia FIGUEREIDO, Juan Pablo ROMERO

**Affiliations:** 1 Faculty of Experimental Sciences, Francisco de Vitoria University, Pozuelo de Alarcón; 2 Brain Injury and Movement Disorders Neurorehabilitation Group (GINDAT), Institute of Life Sciences, Francisco de Vitoria University, Pozuelo de Alarcón; 3 Department of Physiotherapy, Occupational Therapy, Rehabilitation and Physical Medicine, King Juan Carlos University, Alcorcón; 4 Cognitive Neuroscience, Pain, and Rehabilitation Research Group (NECODOR), Faculty of Health Sciences, Rey Juan Carlos University, Madrid, Spain; 5 Institute for Systems and Robotics-Lisboa, Department of Bioengineering, Instituto Superior Técnico, Universidade de Lisboa, Lisbon, Portugal; 6 Neural and Cognitive Engineering group, Centre for Automation and Robotics (CAR) CSIC-UPM, Arganda del Rey, Madrid; 7 Brain Damage Unit, Beata María Ana Hospital, Madrid, Spain

**Keywords:** repetitive transcranial magnetic stimulation, rTMS, motor imagery, neurofeedback, stroke, motor cortex, upper limb

## Abstract

**Objective:**

To examine the clinical effects of combining motor imagery-based neurofeedback training with bilateral repetitive transcranial magnetic stimulation for upper limb motor function in subacute and chronic stroke.

**Design:**

Clinical trial following an AB/BA crossover design with counterbalanced assignment.

**Subjects:**

Twenty individuals with subacute (*n* = 4) or chronic stroke (*n* = 16).

**Methods:**

Ten consecutive sessions of bilateral repetitive transcranial magnetic stimulation alone (therapy A) were compared vs a combination of10 consecutive sessions of bilateral repetitive transcranial magnetic stimulation with 12 non-consecutive sessions of motor imagery-based neurofeedback training (therapy B). Patients received both therapies (1-month washout period), in sequence AB or BA. Participants were assessed before and after each therapy and at 15-days follow-up, using the Fugl-Meyer Assessment-upper limb, hand-grip strength, and the Nottingham Sensory Assessment as primary outcome measures.

**Results:**

Both therapies resulted in improved functionality and sensory function. Therapy B consistently exhibited superior effects compared with therapy A, according to Fugl-Meyer Assessment and tactile and kinaesthetic sensory function across multiple time-points, irrespective of treatment sequence. No statistically significant differences between therapies were found for hand-grip strength.

**Conclusion:**

Following subacute and chronic stroke, integrating bilateral repetitive transcranial magnetic stimulation and motor imagery-based neurofeedback training has the potential to enhance functional performance compared with using bilateral repetitive transcranial magnetic stimulation alone in upper limb recovery.

LAY ABSTRACT

This study evaluated how a combination of 2 non-invasive neuromodulation treatments can help improve arm movement in individuals who have had a stroke. The treatments used were repetitive transcranial magnetic stimulation and mental practice through neurofeedback. Twenty patients participated and were assigned to 1 of 2 groups: either receiving 10 sessions of bilateral repetitive transcranial magnetic stimulation alone, or followed by an additional 12 sessions of neurofeedback during which participants imagined moving the affected arm. The results showed that both groups improved arm function and sensory function. However, the combination of repetitive transcranial magnetic stimulation and mental practice had better results than repetitive transcranial magnetic stimulation alone. Participants experienced a significant improvement in their ability to move and feel with the arm. The effects persisted at least 1 month after completing the treatment. This study shows the potential benefit of using combined neuromodulation therapies in subacute and chronic stages of stroke sequelae.

Globally, an estimated 15 million people experience a stroke each year, many of who exhibit upper limb (UL) motor function deficits within 4 weeks after stroke (1, 2). One semester after the accident, more than 60% of subjects are unable to perform basic activities of daily living (ADLs) due to hand motor deficits (3). Among the primary sensory-motor deficits of the affected UL, loss of handgrip strength, alterations in muscle tone and motor control, and decreased superficial and deep sensitivity significantly impair independence (4).

Conventional UL rehabilitation effectiveness is limited after 3–6 months following acute onset (5). This may be explained by a plateauing of the regain of function during this period; it is likely that the first 3 months correspond to the highest period of circuit plasticity in humans (6).

Repetitive transcranial magnetic stimulation (rTMS) is an exogenous neuromodulation technique that involves applying magnetic stimulation pulses to specific areas of the brain with the purpose of influencing cortical excitability and thus brain plasticity (7). Depending on the frequency of stimulation, this may have an excitatory or inhibitory effect (e.g. frequencies ≤ 1 Hz have an inhibitory effect, while frequencies ≥ 5 Hz increase excitability).

Conversely, neuromodulation can also be performed endogenously. One of the techniques widely validated for this is neurofeedback (NFB), a non-invasive technique that involves real-time monitoring and training of brain activity to improve self-regulation, through providing individuals with real-time information about their brainwave patterns, typically obtained through an electroencephalogram (EEG). This technique is often coupled with motor imagery (MI), providing real-time information about the brain’s activity when imagining motor actions (8).

Both approaches have been used in stroke rehabilitation: rTMS appears to recover strength, increase manual dexterity, and UL functionality, and NFB and MI have been identified as effective strategies for improving motor rehabilitation (9–11).

These neuromodulation techniques have certain limitations, including the potential for short-lived results and significant variability in treatment response among individuals (12). In addition, the majority of efficacy studies conducted on these therapies have utilized small sample sizes, which hinders the generalizability of the results (13, 14).

These techniques are often combined with conventional rehabilitation; however, neuromodulation techniques are not commonly combined with each other. Previous evidence has indicated differences in the scope of neuromodulatory effects between endogenous modulation through MI-based NFB and exogenous modulation via rTMS, with MI-based NFB potentially showing a wider influence on subcortical regions, while rTMS tends to exert its primary impact on cortical areas (15). Therefore, both approaches may have complementary neurophysiological mechanisms to achieve more significant and persistent effects. However, their combination is largely unexamined.

The primary objective of this study was to investigate the clinical effects of adding MI-based NFB training to bilateral rTMS in individuals with stroke. The study hypothesis was that the clinical effects achieved with NFB training after bilateral rTMS would be superior to those of bilateral rTMS alone.

## METHODS

### Study design

A clinical trial following an AB/BA crossover design was conducted following the Consolidated Standards of Reporting Trials (CONSORT) 2010 guidelines (16). The protocol was prospectively registered in clinicaltrials.gov (unique identifier NCT04815486). The study was approved by an independent Clinical Research Ethics Committee at Hospital Universitario de Fuenlabrada, Madrid, following the principles of the Declaration of Helsinki 1964, updated 2013. All participants gave written informed consent before participating. None of the evaluators, therapists, or patients were blinded.

### Participants

**Fig. 1:**
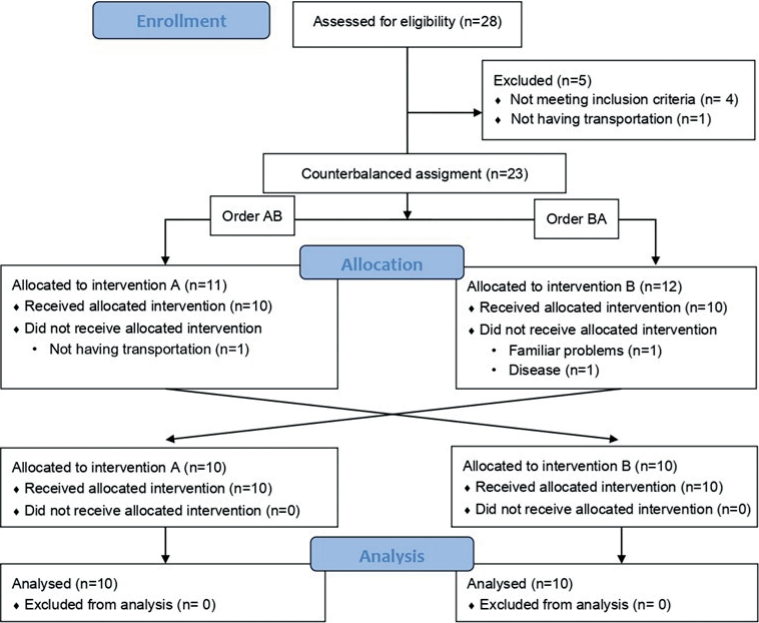
Participant flow diagram according to Consolidated Standards of Reporting Trials (CONSORT) 2010 guidelines.

*Sample size.* Twenty consecutive participants fitting the eligibility criteria (see below) completed the interventions assigned. Fig. 1 shows the participant flow diagram. Four participants in the subacute stage (3–6 months) and 16 participants in the chronic stage (> 6 months) of stroke were mostly recruited from the Brain Injury Unit or Rehabilitation Unit of Hospital Beata María Ana Madrid, Spain. Participants referred from other centres and self-referred patients were also included.

To determine the sample size in this study, various factors were considered. A previous study that utilized rTMS in the rehabilitation of motor sequelae in the UL of patients with chronic stroke has shown an effect size on Fugl-Meyer Assessment (FMA-UL) of Cohen’s d = 1.31 (17). However, that effect size would not be expected in the current study because of: (*i*) the cross-over design, compared with the parallel-group design of the cited study; and (*ii*) the effect of NFB and the rTMS would not be expected to be as large as the previous ones, after accounting for the correlation between repeated measures in the same subjects, and because of the potential ceiling effect reached with the rTMS intervention alone. Therefore, an effect size of Cohen’s d = 0.8 was chosen, considering that a large effect size was expected according to standard interpretation (18), but significantly smaller than that of previous studies, and therefore more conservative. Therefore, an effect size of Cohen’s d = 0.8 was chosen, considering that a large effect size was expected according to standard interpretation (18), but that the expected effect size was significantly smaller than that of previous studies, and therefore more conservative. This calculation was performed in G*Power version 3.1 (Aichach, Germany /www.g-power.de). Accounting for an estimated loss rate of 20%, it was concluded that 23 subjects were needed.

*Eligibility criteria.* The eligibility criteria were as follows. Inclusion criteria: (*i*) age > 18 years, (*ii*) ischaemic or haemorrhagic cerebrovascular injury diagnosed by a neurologist and who have at least 1 brain-imaging test, (*iii*) onset of symptoms > 3 months, (*iv*) presence of upper limb motor sequelae due to stroke; (*v*) stability in anti-spastic medication for more than 5 days, (*vi*) able to read and write, and (vii) sufficient cognitive ability to understand and perform tasks (Token test > 11) (19). Exclusion criteria: (*i*) history of seizure or brain aneurysm, (*ii*) pacemakers, medication pumps, metal implants in the head (except dental implants), (*iii*) clinical instability, (*iv*) aphasia, (*v*) other pre-existing neurological diseases or previous cerebrovascular accidents with sequelae, (*vi*) previous rTMS interventions received after stroke, (*vii*) hemispatial neglect, and (*viii*) flaccid paralysis (Brunnstrom’s stage < 1) (20).

### Interventions

*Bilateral rTMS.* A bilateral rTMS protocol was applied based on the approach of Takeuchi et al. 2009 (21). First, single-pulse TMS was delivered to obtain the resting motor threshold (RMT) using a Magstim Rapid2 device (Magstim Co., Whitland, UK) with a 70-mm figure-of-8 coil. The coil was placed on M1 contralateral to the stroke lesion, and in its homologous on the ipsilesional side as the hot spot was not always identifiable on the lesioned side. RMT was calculated using standardized methods published elsewhere (22).

The rTMS intervention protocol consisted of 10 consecutive daily sessions of bilateral stimulation over a period of 2 weeks (Monday to Friday). Each session comprised stimulation over both M1, starting with low-frequency (1 Hz) stimulation over the contralesional side and, after a resting period of 5 min, high-frequency (10 Hz) stimulation over the lesioned side. Each side received, at 90%RMT (or a maximum intensity of 52% of the default Magstim Rapid2 device maximum output according to its security system), a total of 1000 pulses divided into 20 trains of 50 pulses each, with a 5-s intertrain interval (21).

To assess side-effects, participants were asked at the end of each session whether they experienced tingling, headache or neck pain, drowsiness, and the intensity of these sensations. Safety guidelines for rTMS protocols were followed (23).

*Motor imagery-based neurofeedback.* NeuRow is a MI-based neurofeedback (NFB) training paradigm that allows patients to perform UL motor actions such as they would do in real life. It incorporates a brain-computer interface (BCI) system, which is based on MI and is guided by NFB with EEG.

NeuRow is rendered through a head-mounted virtual reality (VR) headset with a 90° horizontal field of view, and haptic feedback is delivered via 2 controllers in both hands. The paretic limb should be placed in a resting position on the table. After ensuring that the position of the patient and the UL were correct, the EEG cap and the VR viewer were configured. The task consisted of imagining performing unilateral rowing movements with each UL alternately. In the virtual environment, patients saw a boat and 2 high-resolution virtual arms grasping 2 oars in first-person view. The patient had to imagine the movement of each corresponding hand to turn each oar and move forward, observing the imagined movement on the screen. The goal of the task was to perform as many correct motor image sequences as possible in a fixed period.

EEG acquisition was performed using a BCI system with 64 active electrodes equipped with a low noise biosignal amplifier and a 256-Hz 24-bit A/D converter (BrainVision actiCHamp biosignal amplifier, Brain Products GmbH, Gilching, Germany). EEG data were acquired following the international placement of the 10–20 system as follows. The 15 electrodes for BCI were spatially distributed covering mainly the motor and somatosensory areas of the brain. Specifically, Frontal (F3, Fz, F4), Frontal-Central (FC5, FC6), Central (C3, Cz, C4), Central-Parietal (CP5, CP1, CP2, CP6) and Parietal (P3, Pz, P4) electrodes, in a small Laplacian configuration for spatial filtering, were used. Both the processing and the acquisition of the EEG data were carried out with the OpenVibe platform (24), which transmits the data through a Lab Streaming Layer protocol to control the virtual environment.

**Fig. 2:**
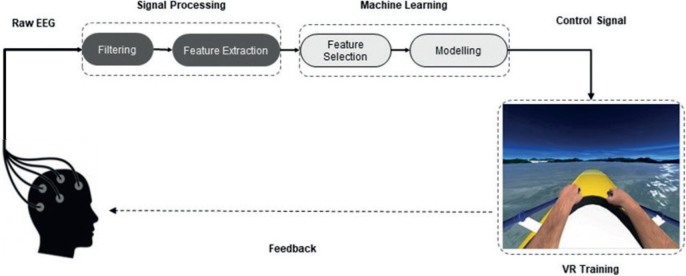
Motor imagery-based neurofeedback with immersive virtual reality (VR) protocol. Adapted from Vourvopoulos et al. Effects of a Brain-computer interface with virtual reality (VR) neurofeedback: a pilot study in chronic stroke patients. Front Hum Neurosci 2019; 13: 210.

A BCI training protocol designed and adapted based on the Graz-BCI paradigm (25) was used. First, acquisition of raw EEG data was performed. Features were extracted in order to train a classifier to distinguish right- and left-hand imaginary movements. This process was carried out by asking the patient to make mental images of the corresponding hand according to the stimuli presented on the screen (Fig. 2). The training session was set up to acquire data in 24 blocks per class (left- or right-hand images) in random order. Subsequently, the data was spatially and temporally filtered between the Alpha and Beta bands (8–30 Hz) to create the feature vector.

The training was carried out in 12 sessions (3 days a week, for 4 weeks) of 30 min each, divided into 3 series of 7 min (26).

### Experimental procedure

The interventions were organized as follows: therapy A consisted in bilateral rTMS protocol exclusively (10 consecutive daily sessions over 2 weeks), whereas therapy B consisted in a combination of the bilateral rTMS protocol and the MI-NFB training. During therapy B the patient received 10 consecutive daily sessions of bilateral rTMS (Monday to Friday, 2 weeks), with the same stimulation parameters as therapy A, and 12 non-consecutive sessions of MI-neurofeedback (3 times a week for 4 weeks). The first 6 MI-NFB sessions were carried out after bilateral stimulation with rTMS (i.e. rTMS as a priming method during the first 2 weeks), and the last 6 sessions, without rTMS as prior priming during the last 2 weeks.

**Fig. 3:**
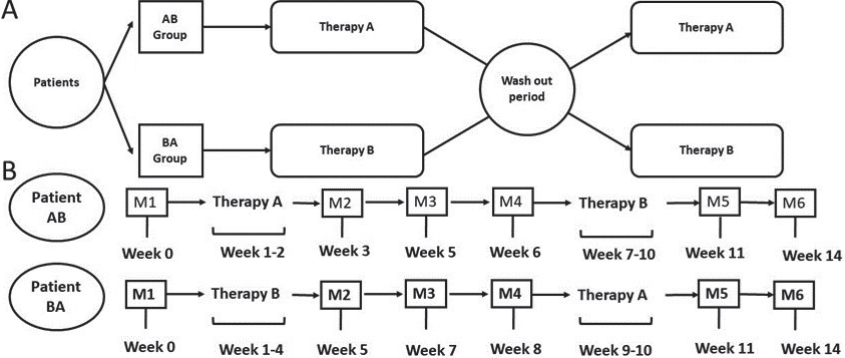
Study design. (A) Therapy sequence in each group, where therapy A is repetitive transcranial magnetic stimulation alone and therapy B is repetitive transcranial magnetic stimulation plus motor imagery-based neurofeedback training. (B) Evaluation timeline. M1–6: measurements.

The clinical trial followed an AB/BA crossover design with counterbalanced assignment, in which the first 50% of the sample was assigned to order AB and the second 50% to order BA. The washout period between therapies A and B was always 1 month (Fig. 3).

Participants were assessed 6 times (M1, M2, M3, M4, M5 and M6), 3 assessments per therapy: an initial evaluation prior to the intervention, a second evaluation in the week after the end of the intervention, and a final evaluation 2 weeks later (Fig. 3).

Throughout the study participation period, patients continued with their usual therapies and neither the dosage of content of these therapies changed during their participation period. These therapies were not homogeneous among participants and included neuropsychology, occupational therapy, physiotherapy, and speech therapy, if needed. None of the therapies was initiated or had changed dosing or frequency during the 2 weeks before inclusion.

All interventions took place in a single laboratory where the temperature was controlled to ensure patient comfort. During sessions, patients were accompanied only by the therapist and were permitted to wear comfortable clothing. To minimize auditory discomfort, ear plugs were provided to patients during the rTMS procedure.

### Primary outcome measures

*Fugl-Meyer Upper Limb Assessment.* The FMA-UL is an observational scale that assesses sensorimotor deficits in post-stroke patients. It includes 4 subscales that make up a total maximum of 66 points. It constitutes the most optimal, sensitive, and appropriate upper limb functionality evaluation scale (27, 28).

*Hand Grip Strength.* An analogue hand dynamometer (Jamar^®^ Plus+ Hand Dynamometer, 0–90 kg; Performance Health Supply, Nottinghamshire, UK) was used to assess isometric grip strength. The patients sat upright, with both feet on the floor and the forearm resting on a stable surface. The patients performed a maximal isometric grip contraction until they reached maximum force production. The mean value of 3 attempts per hand was recorded (29).

*Nottingham Sensory Assessment.* The NSA was used to assess UL somatosensory impairment. It is a discrete quantitative tool that measures, analyses, and interprets the individual’s reactions to a range of sensory stimuli. This scale evaluates tactile sensation, kinaesthetic sensation and stereognosis (30, 31).

### Secondary outcome measures

*Nine-Hole Peg Test.* The 9-HPT assesses fine manual dexterity. The participants were instructed to pick up 9 pegs from a container as quickly as possible and transfer them, 1 by 1, to a pegboard with 9 holes. The test was performed with both hands and the time spent completing the task was noted (32). The 9-HPT is a reliable, valid, and sensitive tool for measuring change among stroke patients (27, 33).

*Computerized Finger Tapping Task.* The FTT test measures motor function and speed of signal processing. Participants sat comfortably in front of a computer and pressed the space bar on the keyboard as quickly as possible and repeatedly with the index finger. Five 10-s attempts were made with the unaffected hand. The mean time between 2 consecutive touches in the 5 attempts was noted (34).

*Arm Motricity Index.* The AMI is a discrete quantitative scale to evaluate muscle strength in 3 actions: pinch, elbow flexion, and shoulder abduction. Each movement is scored, obtaining a total score for the upper limb ranging from 0 (severely affected) to 100 (normal) (35, 36).

### Statistical analysis

Statistical analysis was performed in SPSS 26.0 (SPSS Inc., Chicago, IL, USA) and JASP 0.17.2 (JASP Team 2023). For all analyses, a confidence level of 0.95 (alpha = 0.05) was adopted and 95% confidence intervals (95% CI) were obtained. Prior to hypothesis testing, normality was assured with Shapiro–Wilk test and equality of variances between groups with Levene’s test.

The presence of carry-over effects was first assessed using repeated measures analysis of variance (ANOVA). Therapy effects were assessed using a linear mixed-effects model (LMM) with therapy, time, and sequence (AB/BA) as fixed-effects and participants as random-effects. Although the sample size was initially calculated for a paired samples *t*-test, it was decided to use a LMM, as it allows accommodation of fixed effects considering the inter-subject variability in the model, and therefore is more flexible.

No modifications were made to the methods employed from the inception of the study to its conclusion.

## RESULTS

### Participant characteristics

Patient recruitment spanned a period of 18 months, commencing in September 2021 and concluding in February 2023. The recruitment phase ceased upon reaching the calculated sample size. Participant flow is shown in Fig. 1. From 23 volunteers initially screened, 20 participants completed the study and were finally analysed. Participants’ characteristics at baseline are summarized in Table I and did not differ between groups (*p* > 0.05). Shapiro–Wilk normality test and Levene’s test did not show statistical significance in the outcome measures used (*p* > 0.05). No adverse effects were reported in patients because of either of the therapies administered.

**Table I:** Demographic and clinical characteristics at baseline

Characteristics	AB group (*n* = 10)	BA group (*n* = 10)	*p*-value
Age, years, mean (SD)	68.50 (9.02)	62.30 (8.32)	0.128
Sex, *n* (%)			0.371
Female	6 (60)	4 (40)	
Male	4 (40)	6 (60)	
Time since stroke, months, mean (SD)	27 (21.01)	27.70 (35.51)	0.958
Stroke type, *n* (%)			0.606
Ischaemic	7 (70)	8 (80)	
Haemorrhagic	3 (30)	2 (20)	
Affected structure, *n* (%)			0.541
Cortical	6 (60)	7 (70)	
Subcortical	4 (40)	3 (30)	
Dominance, *n* (%)			0.305
Right	10 (100)	9 (90)	
Left	0 (0)	1 (10)	
Affected hemisphere, *n* (%)			0.178
Right	3 (30)	6 (60)	
Left	7 (70)	4 (40)	
Montreal Cognitive Assessment, mean (SD)	24.16 (5.08)	23.40 (3.97)	0.115
Fugl Meyer Assessment-Upper Limb, mean (SD)	24.50 (16.21)	24.50 (11.84)	0.513
Hand-grip strength, kg, mean (SD)	4.03 (4.34)	4.87 (4.73)	0.677
Nine Hole Peg Test, s, mean (SD)	167.39 (94.92)	180.75 (103.76)	0.626
Arm Motricity Index, mean (SD)	42.10 (21.60)	42.40 (22.64)	0.852
Finger Tapping Test, s, mean (SD)	586.05 (802.16)	1921.54 (2803.82)	0.206
Nottingham Sensory Assessment-Tactile Sensations, mean (SD)	1.49 (0.57)	1.34 (0.36)	0.947
Nottingham Sensory Assessment-Kinesthetic Sensations, mean (SD)	1.30 (0.53)	1.75 (0.48)	0.051
Nottingham Sensory Assessment-Stereognosis, mean (SD)	4.49 (3.89)	5.16 (4.05)	0.674

### Primary outcome measures

The different values obtained for each outcome measure and time of measurement are shown in Table II.

**Table II:** Values of outcome measures by time and sequence of treatment

Variable	Measurement					
	1	2	3	4	5	6
FMA-UL, mean (SD)						
AB	24.50 (16.21)	29.20 (18.13)	29.20 (17.35)	28.80 (16.59)	36.50 (16.41)	36.70 (16.64)
BA	24.50 (11.84)	42.50 (18.51)	42.50 (19.50)	42.20 (19.16)	43.50 (18.78)	42.90 (20.53)
Hand-grip strength, kg, mean (SD)						
AB	4.03 (4.34)	4.47 (4.31)	4.16 (4.37)	4.03 (4.10)	5.18 (4.74)	5.04 (4.37)
BA	4.87 (4.73)	9.10 (10.47)	7.18 (6.13)	6.54 (6.11)	7.13 (6.21)	7.44 (7.39)
NSA-TS, mean (SD)						
AB	1.49 (0.57)	1.45 (0.60)	1.36 (0.57)	1.30 (0.55)	1.77 (0.48)	1.76 (0.42)
BA	1.34 (0.36)	1.76 (0.38)	1.74 (0.39)	1.77 (0.69)	1.66 (0.59)	1.70 (0.50)
NSA-KS, mean (SD)						
AB	1.30 (0.53)	1.35 (0.47)	1.35 (0.47)	1.22 (0.47)	2.12 (0.77)	1.98 (0.52)
BA	1.75 (0.48)	2.30 (0.42)	2.37 (0.56)	2.27 (0.55)	2.17 (0.60)	1.95 (0.43)
NSA-S, mean (SD)						
AB	4.49 (3.89)	4.50 (3.89)	4.40 (3.97)	4.40 (3.97)	4.63 (3.77)	4.53 (3.86)
BA	5.16 (4.05)	5.46 (3.72)	4.76 (3.64)	4.63 (3.76)	4.70 (3.71)	4.76 (3.64)
9-HPT, s, mean (SD)						
AB	167.39 (94.92)	149.23 (91.20)	137.52 (93.14)	153.02 (106.54)	138.86 (99.65)	124.64 (96.33)
BA	180.75 (103.76)	151.04 (81.72)	167.72 (103.40)	171.71 (139.92)	146.27 (121.17)	107.75 (91.84)
AMI, mean (SD)						
AB	42.10 (21.60)	50.80 (22.30)	48.50 (25.35)	40.40 (24.51)	65.70 (26.00)	64.10 (29.18)
BA	42.40 (22.64)	70.70 (23.99)	67.70 (29.22)	66.20 (28.06)	70.80 (23.60)	70.00 (24.50)
FTT, ms, mean (SD)						
AB	586.05 (802.16)	520.14 (640.96)	633.13 (649.71)	708.34 (840.04)	2,827.66 (6,893.94)	670.66 (598.86)
BA	1,921.54 (2,803.82)	2,734.07 (3,700.31)	1,738.35 (3,156.39)	1,536.39 (3,076.39)	514.58 (464.81)	469.29 (674.84)

Arm MI: Arm Motricity Index; FMA-UL: Fugl-Meyer Assessment-Upper Limb; FTT: Finger Tapping Test; 9-HPT: Nine-Hole Peg Test; NSA-TS: Nottingham Sensory Assessment-Tactile Sensations; NSA-KS: Nottingham Sensory Assessment-Kinesthetic Sensations; NSA-S: Nottingham Sensory Assessment-Stereognosis; SD: standard deviation.

**Fig. 4:**
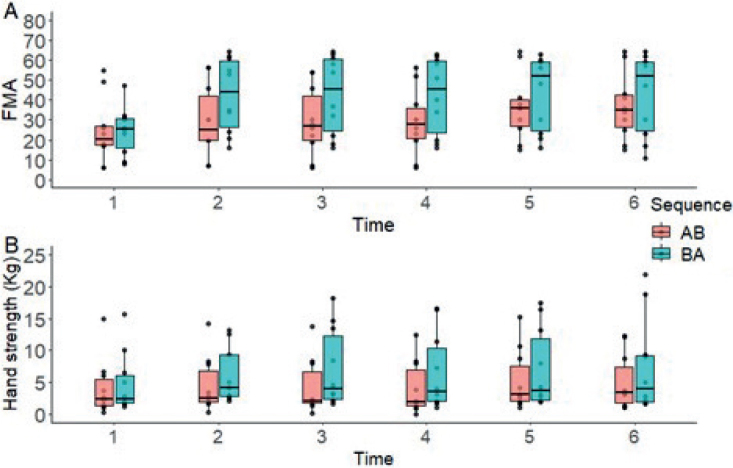
Changes in (A) Upper Limb Fugl-Meyer Assessment (FMA-UL) and (B) Hand-Grip Strength (Kg) over time, according to the treatment sequence.

*Fugl-Meyer Upper Limb Assessment.* FMA-UL was the only variable that showed a significant carry-over effect (*p* = 0.03). LMM showed significant effects for the therapy factor for FMA-UL (F (1, 64) = 27.096; *p* < 0.001) and factor time (F (5, 90) = 39.246; *p* < 0.001). No effects were found for the factor sequence (F (1, 18) = 1.324; *p* > 0.05). Significant effects were found for sequence by time (F (5, 90) = 9.164; *p* < 0.001). Post-hoc analyses showed significant differences at measurement 2 (E = 4.433; t = 5.385; *p* < 0.001), 3 (E = –2.217; t = –2.692; *p* = 0.008), 4 (E = –2.217; t = –2.692; *p* = 0.008) and 5 (E = –2.267; t = –2.753; *p* = 0.007) in favour of therapy B (Fig. 4A). No other measurements showed significant differences (*p* > 0.05).

*Hand Grip Strength.* The LMM did not show significant outcomes for the therapy factor (F (1, 18) = 0.311; *p* > 0.05) and the sequence factor (F (1, 18) = 1.053; *p* > 0.05) concerning Hand Grip Strength. However, significant effects were observed for the time factor (F (5, 90) = 3.127; *p* < 0.001). A significant interaction between sequence and time was not detected (F (5, 90) = 1.823; *p* > 0.05). Subsequent post-hoc analyses showed significant differences at measurement 2 (E = 0.862; t = 2.030; *p* = 0.045) and 3 (E = –1.036; t = –2.440; *p* = 0.017) favouring therapy B (Fig. 4B). No significant variations were observed in the remaining measurements (*p* > 0.05).

### Nottingham Sensory Assessment (NSA)

**Fig. 5:**
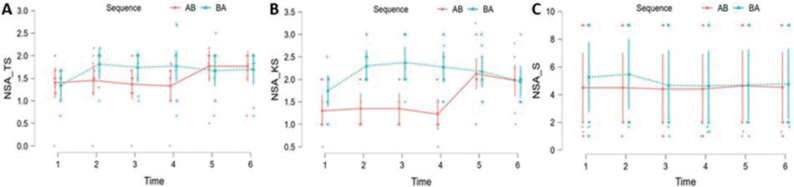
Changes in Nottingham Sensory Assessment (NSA): (A) Tactile Sensation (TS), (B) Kinesthetic Sensation (KS) and (C) Stereognosis (S) over time, according to the treatment sequence.

- *Tactile Sensation (NSA-TS).* Significant effects were observed for the therapy factor (F (1, 18) = 7.065; *p* = 0.016), as well as for the time factor (F (5, 90) = 7.130; *p* < 0.001). No significant effects were found for the sequence factor (F (1, 18) = 0.557; *p* > 0.05). A significant interaction effect was observed between sequence and time (F (5, 90) = 6.759; *p* < 0.001). There were significant differences at all measurements (*p* < 0.05) in favour of therapy B (Fig. 5A).
- *Kinesthetic Sensation (NSA-KS).* Significant effects were observed for the therapy factor (F (1, 18) = 9.036; *p* = 0.004), as well as for the time factor (F (5, 90) = 11.146; *p* < 0.001) and sequence factor (F (1, 18) = 7.647; *p* = 0.013). A significant interaction effect was observed between sequence and time (F (5, 90) = 15.557; *p* < 0.001). There were significant differences at all measurements (*p* < 0.05) in favour of therapy B except at time 2 (E = 0.066; t = 1.156; *p* > 0.05) (Fig. 5B).
- *Stereognosis (NSA-S).* No significant effects were observed for the therapy factor (F (1, 18) = 0.636; *p* > 0.05), the time factor (F (5, 90) = 1.009; *p* > 0.05), sequence factor (F (1, 18) = 0.063; *p* > 0.05), as well as for sequence and time interaction (F (5, 90) = 0.905; *p* > 0.05). There were no significant differences at any measurements (*p* > 0.05) (Fig. 5C).

### Secondary outcome measures

The therapy factor showed significant effects on the AMI (F (1, 93) = 13.021; *p* < 0.001). The sequence factor had no significant effect on the changes produced by therapies in any outcome measure (*p* > 0.05). Both AMI and 9-HPT exhibited significant effects for the time factor (F (5, 90) = 17.556; *p* < 0.001) and (F (5, 65) = 3.097; *p* = 0.014), respectively). Regarding the interaction between the sequence and time factors, significant effects were only found in AMI (F (5, 90) = 4.949; *p* < 0.001). Only measurements 1 (E = 2.300; t = 2.953; *p* = 0.004) and 2 (E = –6550; t = –3120; *p* = 0.002) showed significant changes in favour of therapy B. No significant effects were found for any of the factors on the FTT.

## DISCUSSION

This study investigated whether the combined application of bilateral rTMS and MI-based NFB training improves UL motor function to a greater extent than rTMS alone in subacute and chronic post-stroke patients. A significant improvement was observed in UL motor function (FMA-UL), favouring the combined protocol (rTMS + MI-based NFB). However, contrary to expectations, hand-grip strength did not show significant between-therapy differences. For the somatosensory deficits (NSA), improvements were notable particularly in tactile and kinaesthetic sensation, again favouring the combined therapy, although without differences in stereognosis. In addition, the combined approach also had a positive impact on secondary outcome measures, such as AMI. Altogether, these results support the hypothesis of the study.

The FMA-UL was the only variable that presented a significant carry-over effect, suggesting that the effects of rTMS were maintained beyond the intervention period. As all participants continued their conventional rehabilitation therapies during the washout month, it cannot be ruled out that this could have influenced the permanence of the effects.

Beneficial effects of combining rTMS and BCI training have been proved in previous studies; our findings confirm this idea in general, but also provides a broader understanding of clinical efficacy of this specific combination. One such study, by Johnsson et al. (37), compared a 2-week combined protocol of rTMS and BCI training against sham-rTMS in a cross-over design (*n* = 3). Their methods diverged from ours in terms of rTMS parameters (wherein they utilized low frequency on the unaffected side, at 1 Hz and 90% RMT for 10 min over 9 sessions, in contrast to our 10-session bilateral protocol) and a variant form of BCI training (centred on opening and closing of the hand throughout 18 sessions, with the initial 9 primed by rTMS and the remaining 9 without). Their outcomes showed significant enhancements in gross manual dexterity (Box and Blocks test) in the sham rTMS+BCI condition and in both conditions for the FTT test on the affected side proving a beneficial effect of BCI, but with a clear enhancement by rTMS. In a similar way to Johnsson the current study also proved the superiority of the combination of both therapies based on improvement of functional scales (FMA-UL), but the current study protocol additionally showed effects on sensory variables.

Conversely, Chen et al. (38) used iTBS on the affected hemisphere as a primer to VR cycling training for UL compared with sham iTBS as the priming for the same training. This study confirmed the beneficial effects of VR, but a clear enhancement by the iTBS priming was also evident in Modified Ashworth Scale Upper-Extremity, Motor Activity Log and Stroke Impact Scale. Unlike the current study, although Chen et al. (38) showed improvement for FMA-UL, they did not demonstrate significant between-group differences.

Despite the methodological differences, these 2 studies suggest that pre-training rTMS stimulation can modify or enhance the effects of NFB or VR. In a complementary manner, the current study demonstrates that MI-based NFB enhances the effect of bilateral rTMS. The optimal type of rTMS stimulation and the therapy to be combined to, however, remains a topic of debate. Bilateral rTMS application relies on the combined neurophysiological effects derived from both the decreased excitability on the unaffected side (37) and the increased excitability on the affected hemisphere (38).

In the current study, the absence of substantial differences between the therapies and sequence in hand-grip strength implies that both therapeutic strategies are comparably effective in enhancing this metric. It is worth noting that the NFB is based in imagined movement, which is congruent with the observation that, in the NSA, the combined approach showed notable effects on the touch and movement sensation tests (i.e. positive changes in the distal part of the limb), improving both skills. The effectiveness of this strategy in enhancing sensory components, may be attributed to the vibrating touch feedback and the embodiment that NeuRow provides (39, 40).

In terms of secondary variables, a significantly different effect between therapies was observed only in the AMI. This supports, along with the FMA-UL results, the clinical efficacy of the combined approaches in enhancing the functionality of the impaired limb. The 9-HPT showed notable time effects, indicating that patients experienced significant improvements in fine manual dexterity after receiving both therapies, with no discernible differences between them. One possible explanation to this lack of adjuvant effect of MI based- NFB could be a ceiling effect already achieved by rTMS stimulation. We hypothesize that functional brain changes for improving reaching movements involving the shoulder/elbow and distal motor control could rely on different stages of recovery or diverse neural plasticity patterns driving functional recovery (41), which may explain why enhancement of distal improvements in motor components were not observed with the combined treatment. Lastly, neither the brain changes induced by any of the interventions appeared to alter participants’ brain processing speed, measured with a FTT paradigm on the unaffected side. This could imply a ceiling effect for FTT on the unaffected hemisphere, but also implies that bilateral rTMS did not disrupt the function of the non-affected hemisphere in terms of FTT and possibly cognitive processing speed.

### Limitations

This study has some limitations. First, it is important to acknowledge that the study was not conducted using a randomized double-blind approach. While the results are certainly significant and important, caution is required when extending these results to broader contexts. Secondly, there was a difference in the duration of the rTMS and NFB neuromodulation protocols (10 sessions in 2 weeks and 12 sessions in 4 weeks, respectively). This discrepancy, which is due to the design of the study based on previous neuromodulation studies (21, 26), could have affected the results. The assessments were carried out immediately after the whole intervention finished. This might have missed midterm changes of the rTMS treatment for the group receiving therapy A, or immediate effects from rTMS in the therapy B group.

The absence of corrections for multiple comparisons poses a potential limitation, which might affect the risk of obtaining false-positive results. In addition, given that each participant underwent the evaluation battery 6 times, the study did not account for potential learning effects, which might influence the outcomes across repeated assessments.

Finally, the participants continued with their usual neurorehabilitation routine during the treatment, which was not controlled in this research. However, it is important to note that these routines remained consistent for each participant throughout the study period, minimizing their impact on the study of treatment effects due to the crossover design.

This study demonstrates several key findings. MI-based NFB can amplify the impact of bilateral rTMS on the functional evaluation and somatosensory perception of the upper limb, boosting the effects of conventional therapy. Nonetheless, this interaction may also be indicative of a potential priming effect of rTMS on NFB efficacy. This finding supports the previous reports of adjuvant effects of rTMS to other therapeutic interventions for post-stroke motor recovery.

Although the study was not explicitly structured to assess the persistence of the effects of the interventions, the observed carryover effect suggests a lasting effect of at least 1 month after bilateral rTMS intervention in both groups.

There is a differential impact of MI-based NFB on UL functionality potentiation vs strength, which may indicate a specific effect of motor imagery add on in contrast with other strategies used in different studies with also different results. This reinforces the need for personalized therapy plans.

The study’s objective was not to investigate the safety of bilateral rTMS stimulation with injured hemisphere stimulation and unaffected hemisphere inhibition. However, the current findings confirm that it is a safe procedure. There was no decline in function due to inhibition of the unaffected hemisphere, nor were there adverse effects from stimulating the affected side.

### Conclusion

These findings suggest that rTMS is a promising treatment for the rehabilitation of stroke patients in both subacute and chronic phases. Combining rTMS with other non-invasive neuromodulation strategies, such as neurofeedback (NFB) and virtual reality (VR), should be tailored to target specific rehabilitation goals, whether to improve dexterity, sensitivity, or strength. The use of these combined therapies in clinical settings could significantly impact a large subset of patients, particularly where therapeutic options are limited, as in the subacute and chronic phases. Further validation of the trends observed in this exploratory study is necessary, using a randomized clinical trial.
